# Meta-research metrics matter: letter regarding article “indirect tolerability comparison of Deutetrabenazine and Tetrabenazine for Huntington disease”

**DOI:** 10.1186/s40734-017-0067-x

**Published:** 2017-11-22

**Authors:** Filipe B. Rodrigues, Gonçalo S. Duarte, João Costa, Joaquim J. Ferreira, Edward J. Wild

**Affiliations:** 1 Huntington’s Disease Centre, Institute of Neurology, University College London, Russell Square 10-12, London, WC1B 5EH UK; 2 Laboratory of Clinical Pharmacology and Therapeutics, Faculty of Medicine, University of Lisbon, Lisbon, Portugal; 3 Clinical Pharmacology Unit, Instituto de Medicina Molecular, Lisbon, Portugal; 4 Cochrane Movement Disorders Group, Faculty of Medicine, University of Lisbon, Lisbon, Portugal; 5 Center for Evidence-Based Medicine, Faculty of Medicine, University of Lisbon, Lisbon, Portugal

**Keywords:** Huntington’s disease, Tetrabenazine, Deutratrabenazine, Indirect treatment comparison

## Abstract

Here we discuss the report by Claassen and colleagues describing an indirect treatment comparison between tetrabenazine and deutetrabenazine for chorea in Huntington’s disease using individual patient data. We note the potential for discrepancies in apparently statistically significant findings, due to the rank reversal phenomenon. We provide some cautionary observations and suggestions concerning the limitations of indirect comparisons and the low likelihood that good quality evidence will become available to guide clinical decision comparing these two agents.

To the Editor,

We read with interest the report by Claassen and colleagues describing an indirect treatment comparison between tetrabenazine (TBZ) and deutetrabenazine (DEU) for chorea in Huntington’s disease (HD) using individual patient data [1].

DEU is a form of TBZ, chemically-modified to optimize its pharmacokinetic properties. Both are vesicular monoamine transporter 2 (VMAT2) inhibitors and each was tested successfully against placebo for chorea associated with HD [2, 3]. No double-blinded head-to-head comparison has been performed or is planned to compare the efficacy and safety profiles of these compounds.

Indirect treatment comparisons are useful meta-research tools when little or no data directly comparing treatment are available [4]. In meta-research, the use of individual patient data instead of aggregate data has many potential advantages, such as the power to study subgroups and to control for confounding factors. To some extent Claassen et al. used individual patient data, since raw patient data from the FIRST-HD trial testing DEU was incorporated into the analysis. This partially overcomes possible reporting bias from the literature, such as adverse events frequencies not recorded in primary reports, and provides the opportunity to use more complex and complete statistical models adjusted to important covariates. It is a shame that individual patient data from the TETRA-HD trial [3] were not included, especially since both trials were performed by the same study consortium (the Huntington Study Group). In addition, it would have been both possible and interesting to undertake exploratory analyses to find out whether subgroups of patients with different genders, CAG repeat lengths, baseline levels of functional ability, motor symptoms and quality of life differed in regards to safety profile.

We also think it unfortunate that the authors limited their report to safety data, while it would be extremely relevant to learn how the efficacy profiles of TBZ and DEU compared using the individual patient data available to them. We recently performed an indirect treatment comparison using all published aggregate data from the same trials, and found no difference between the primary efficacy outcomes of both trials: the total maximal chorea score change from baseline mean difference was between TBZ and DEU was −1.00 (95% confidence interval: −3.04 to 1.04) [5].

Interestingly, in contrast to Claasen and co-authors’ findings, our safety outcomes did *not* demonstrate any difference between odds ratios of TBZ over DEU (Table 1). However, when converting our raw dataset to risk differences instead of odds ratios, as presented by Claassen et al., our results were in line with theirs, in regards to direction and magnitude of effect. This is a well-described statistical phenomenon called *rank reversal*. It stems from the fact that different measures (e.g. risk differences, risk ratios, and odds ratios) are affected differently by dissimilar baseline risks [6]. Bucher’s model of indirect treatment comparisons was originally designed for odds ratios, but others have applied it to risk ratios and risk differences, with proper adjustment.

**Table 1:** Indirect treatment comparison between TBZ and DEU (as reported by us in Rodrigues et al. [5]), both in odds ratios as initially reported, and converted to risk differences; and as reported by Claassen et al. [1] as risk differences

Adverse event	Indirect comparison		
	Rodrigues et al. [5]		Claassen et al. [1]
	OR (95% CI) (TBZ vs DEU)	RD (95% CI) (DEU vs TBZ)	RD (95% CI) (DEU vs TBZ)
SAE	5.44 (0.09 to 322.08)	−0.07 (−0.17 to 0.03)	−0.074 (−0.167 to 0.019)
Somnolence	4.95 (0.34 to 72.37)	−0.21 (−0.39 to −0.03)*	−0.215 (−0.392 to −0.037)*
Diarrhoea	0.07 (0.03 to 2.06)	0.12 (−0.03 to 0.27)	0.115 (−0.038 to 0.268)
Insomnia	14.18 (0.47 to 426.77)	−0.24 (−0.40 to −0.04)*	−0.237 (−0.387 to −0.087)*
Fatigue	1.21 (0.31 to 11.14)	−0.07 (−0.26 to 0.12)	−0.067 (−0.256 to 0.123)
Falls	2.71 (0.31 to 23.98)	−0.07 (−0.26 to 0.12)	−0.078 (−0.265 to 0.110)
Depression	17.15 (0.55 to 531.90)	−0.17 (−0.31 to −0.28)*	−0.170 (−0.304 to −0.037)*

Presentation as risk differences produces statistically significant differences between DEU and TBZ that are not seen when presented as odds ratios; neither approach is intrinsically more accurate and an awareness of the difference is important. TBZ, tetrabenazine; DEU, deutetrabenazine; OR, odds ratio; 95% CI, 95% confidence interval; RD, risk difference; SAE, severe adverse events; *p-value < 0.05

When indirectly comparing treatments, the choice of presented metric matters. We believe it is important for readers to be made aware of the potential for apparently discrepant findings that may arise from the rank reversal phenomenon [6]. Undoubtedly, risk differences increase interpretability, but odds ratios are the only measure that guarantees the avoidance of impossible predicted event rates when extrapolating the results for real populations (for example, applying a risk difference of 0.1 to a population with a risk of 0.05 would give rise to an apparent population risk with a value less than zero, which is implausible) and relative measures are known to be more consistent than absolute measures [7–9].

Choice of data and outcomes aside, an overarching concern is that none of the included clinical trials has been appropriately powered to investigate the safety of these compounds, and an indirect treatment comparison of one trial per agent (i.e. TBZ versus placebo, and DEU versus placebo), cannot improve the precision of the results. Therefore, the results of these comparisons [1, 5] should be interpreted with caution; and although regarded as best *available* evidence, they are nonetheless of low quality according to the Grading of Recommendations Assessment, Development and Evaluation (GRADE) framework [10].

Given all this, only a direct comparison between TBZ and DEU would be able to rigorously test whether the efficacy profiles of these compounds significantly differ, and our sample size calculation (over 600 participants) suggests that such a trial is unlikely to take place [5]. Further post-authorisation safety studies or other observational studies will be required to provide robust evidence on the safety profile of DEU to inform safe prescribing decisions.

## Abbreviations

DEU: deutetrabenazine
GRADE: Grading of Recommendations Assessment, Development and Evaluation
HD: Huntington’s disease
TBZ: tetrabenazine
VMAT2: vesicular monoamine transporter 2

## References

[1] Claassen DO, Carroll B, De Boer LM, Wu E, Ayyagari R, Gandhi S, Stamler D (2017). Indirect tolerability comparison of Deutetrabenazine and Tetrabenazine for Huntington disease. J Clin Mov Disord.

[2] Huntington Study Group (2006). Tetrabenazine as antichorea therapy in Huntington disease: a randomized controlled trial. Neurology.

[3] Huntington Study Group (2016). Effect of Deutetrabenazine on chorea among patients with Huntington disease: a randomized clinical trial. JAMA.

[4] Bucher HC, Guyatt GH, Griffith LE, Walter SD (1997). The results of direct and indirect treatment comparisons in meta-analysis of randomized controlled trials. J Clin Epidemiol.

[5] Rodrigues FB, Duarte GS, Costa J, Ferreira JJ, Wild EJ: Tetrabenazine versus deutetrabenazine for Huntington's disease: twins or distant cousins? Mov Disord Clin Pract 2017:n/a-n/a. 10.1002/mdc3.12483 PMC5573977 28920068

[6] Norton EC, Miller MM, Wang JJ, Coyne K, Kleinman LC (2012). Rank reversal in indirect comparisons. Value Health.

[7] Walter SD (2000). Choice of effect measure for epidemiological data. J Clin Epidemiol.

[8] Deeks JJ (2002). Issues in the selection of a summary statistic for meta-analysis of clinical trials with binary outcomes. Stat Med.

[9] Engels EA, Schmid CH, Terrin N, Olkin I, Lau J (2000). Heterogeneity and statistical significance in meta-analysis: an empirical study of 125 meta-analyses. Stat Med.

[10] Guyatt GH, Oxman AD, Schunemann HJ, Tugwell P, Knottnerus A (2011). GRADE guidelines: a new series of articles in the journal of clinical epidemiology. J Clin Epidemiol.

